# Response of a Habitat-Forming Marine Plant to a Simulated Warming Event Is Delayed, Genotype Specific, and Varies with Phenology

**DOI:** 10.1371/journal.pone.0154532

**Published:** 2016-06-03

**Authors:** Laura K. Reynolds, Katherine DuBois, Jessica M. Abbott, Susan L. Williams, John J. Stachowicz

**Affiliations:** 1 Department of Evolution and Ecology, University of California Davis, Davis, CA 95616, United States of America; 2 Bodega Marine Lab, University of California Davis, Bodega Bay, CA 94923, United States of America; University of Connecticut, UNITED STATES

## Abstract

Growing evidence shows that increasing global temperature causes population declines and latitudinal shifts in geographical distribution for plants living near their thermal limits. Yet, even populations living well within established thermal limits of a species may suffer as the frequency and intensity of warming events increase with climate change. Adaptive response to this stress at the population level depends on the presence of genetic variation in thermal tolerance in the populations in question, yet few data exist to evaluate this. In this study, we examined the immediate effects of a moderate warming event of 4.5°C lasting 5 weeks and the legacy effects after a 5 week recovery on different genotypes of the marine plant *Zostera marina* (eelgrass). We conducted the experiment in Bodega Bay, CA USA, where average summer water temperatures are 14–15°C, but extended warming periods of 17–18°C occur episodically. Experimental warming increased shoot production by 14% compared to controls held at ambient temperature. However, after returning temperature to ambient levels, we found strongly negative, delayed effects of warming on production: shoot production declined by 27% and total biomass decreased by 50% relative to individuals that had not been warmed. While all genotypes’ production decreased in the recovery phase, genotypes that grew the most rapidly under benign thermal conditions (control) were the most susceptible to the detrimental effects of warming. This suggests a potential tradeoff in relative performance at normal vs. elevated temperatures. Modest short-term increases in water temperature have potentially prolonged negative effects within the species’ thermal envelope, but genetic variation within these populations may allow for population persistence and adaptation. Further, intraspecific variation in phenology can result in maintenance of population diversity and lead to enhanced production in diverse stands given sufficient frequency of warming or other stress events.

## Introduction

Over the next century, mean temperatures are expected to increase [1]; furthermore, increased temperature variability is likely to result in a more frequent short-term, extreme warming events [2,3]. Plant populations are already responding to these shifts in temperature. In many species, geographic ranges are shifting in latitude and in elevation towards cooler regions [4,5], and some of these episodic warm events have resulted in large population reductions in regions where species are living close to their thermal tolerances [6,7]. In regions where temperatures are well within tolerable limits, warming temperatures can increase species’ vital rates [8], but the long-term consequences of these responses for an individual energy balance, long-term growth, and the dynamics of populations is often unclear. Incorporating populations that live in environments distant from their thermal limits into climate change studies is rare.

Through a variety of mechanisms, standing genetic variation can impact population response to episodic disturbances. Selection for individuals with tolerance to extreme conditions is one obvious mechanism for resistance to disturbance [9,10,11]. Other studies show that facilitation or niche complementarity among individuals can play a significant role in maintaining population size [12,13]. For example, in the face of environmental fluctuations, genetically based variability in life history strategies increase salmon population size and thus fishery catch stability [14]. Likewise, individuals of the pasture grass, *Anthoxanthum odoratuum*, were more likely to survive aphid infestation if surrounded by unrelated individuals [15]. Still another possibility is that phenotypic plasticity (or acclimation) may play an important role in population resilience. Plants that either exhibit tolerance to stressful environmental conditions or induce changes in morphology or physiology that allow them to maintain high fitness should be more likely than other individuals to persist as stressors increase [16,17].

In a world in which environmental conditions are changing rapidly in ways that depart from long-term means, understanding how intraspecific genetic diversity allows populations to adapt to changing environmental stressors is essential for effective conservation and management of resources. For example, typical goals of an ecosystem restoration are to quickly establish plants that will persist long-term, and provide a full suite of ecosystem services including habitat provision, secondary production, and the cycling of materials and energy. Balancing these goals and choosing the correct combinations of source material relies on a good understanding of how plant traits change in response to environmental variation and how genotypes vary in their response to a fluctuating environment [18].

In this study, we assess how eelgrass (*Zostera marina*) genotypes vary in their response to a simulated summer heat wave. These clonal plants typically grow in monospecific meadows that provide a suite of important ecosystem services including carbon sequestration, nutrient filtration, habitat, and sediment stabilization [19]. Seagrasses are sensitive to environmental changes and are showing rapid decline worldwide, in part due to anthropogenic disturbances [20,21]. Meadows with increased genetic diversity provide more ecosystem services both under stressful [12,13,22] and apparently unstressful conditions [23]. Individual plant traits do vary among genotypes in common gardens [24,25], but the extent to which individual plants (and their traits) respond differently to stressors is not as well understood (but see [25,26]).

A growing number of studies are finding that in some regions temperature is an important driver of eelgrass decline [7,27]. Our study was conducted using plants from Bodega Bay, CA USA where temperatures are moderate—with average temperatures around 14–15°C [28]. Given the broad distribution of eelgrass meadows in the northern hemisphere (from polar regions to mid-latitudes that generally stay below 30°C [29]), this climate is relatively mild. We used a realistic manipulation of temperature, maintaining diurnal fluctuations but increasing the mean by 4.5°C. This happens occasionally in this region during summer periods of reduced fog, relaxation of coastal upwelling, or neap tides that reduce exchange between the bays and estuaries where eelgrass lives and the cooler coastal ocean [28]. Both during the warming event and after a recovery period of 3–5 weeks, we monitored growth, biomass allocation and other morphological and physiological traits of seven eelgrass genotypes.

## Methods

### Growing conditions and temperature manipulation

We grew seven eelgrass genotypes in 16 experimental outdoor mesocosms with flow-through seawater. Half of the mesocosms were subjected to a simulated heat wave that lasted 5 weeks. We measured growth and traits related to production under both elevated and ambient (control) temperatures before the temperature treatment was applied (acclimation), at the end of the elevated temperature period (treatment), and after the plants had several weeks to recover at ambient temperatures (recovery).

A single mesocosm consisted of a 120 L tank (60 cm x 30 cm x 66 cm deep) supplied with continuously flowing, coarsely filtered seawater. We placed twenty-one 8.9 x 8.9 cm plastic pots filled with coarsely sieved local sediment within each mesocosm. We randomly assigned each pot to one of seven genotypes (G1–G7) and we sacrificed one pot of each genotype at each of three time periods (acclimation, treatment, and recovery—described below). We chose these genotypes haphazardly from a set of 42 collected from native Bodega Bay meadows currently growing in culture at Bodega Marine Lab [30] such that they were representative of the trait variation present in the full set of 42. In each pot, we planted a single ramet standardized to a maximum leaf length of 30 cm and a rhizome length of 3.0 cm by gently pushing the rhizome into the sediment. We supplied half of the mesocosms with natural seawater at ambient temperature (control), and half with ambient water that had been passed through a header tank with titanium heaters (elevated). We randomly assigned each mesocosm to a temperature treatment (control vs elevated). All mesocosms had a flow rate of approximately 0.8–1.0 L min^-1^. We recorded the temperature in each mesocosm every 15 min using HOBO data loggers. We measured nutrient content of the water eight times spaced over the course of the experiment, by collecting triplicate 30 mL water column samples, passing them through a 0.45 μm glass fiber filter, and freezing until nitrate concentration could be analyzed using a Lachat 8000 series flow injection auto analyzer.

Plants in both control and elevated temperatures acclimated to the mesocosm at ambient seawater temperature for five weeks after planting (acclimation phase). We then exposed half of the mesocosms to heated (+ 4.5C over ambient) water for five weeks (treatment). Finally, we returned all mesocosms to ambient temperature for another five weeks (recovery). During the final two weeks of each of these time periods, we sacrificed one ramet of each genotype to assess plant production and traits, including chlorophyll a fluorescence, plant morphology and chemistry, growth rate, and nutrient uptake. The duration of the treatments thus varied somewhat among replicates due to the time required to measure traits on so many individuals, but we interspersed sampling of ramets from elevated and control temperatures such that mean time until harvest was the same for plants from both temperatures.

### Quantifying plant traits and production

We used Pulse Amplitude Modulation (PAM) fluorometry to measure chlorophyll fluorescence [DIVING-PAM (Walz, Germany)] for each terminal shoot in the outdoor mesocosms. We placed the manufacturer’s 4 mm diameter dark leaf clip 20 cm from the sediment surface on an outer leaf cleaned of epiphytes. After a 30 min dark acclimation, we determined quantum yield using the light saturation method (F_V_/F_m_) as a proxy for stress, and a rapid light curve (RLC) was performed to assess light adaptation. The RLC was composed of actinic light applied in 8 incremental steps from 0 to a maximum ranging from 2550–3500 μmol photons m^− 2^ s^− 1^, and the resulting yield measurements were converted to electron transport rates using the equation:

$$ETR_{MAX}=Yield\times PAR\times 0.5\times AF$$

where AF is the standard absorption factor (0.55) of a seagrass leaf [31,32], and 0.5 assumes that photons absorbed are equally distributed between photosystems I and II [33]. We fit the data to a double exponential decay function [34] from which we calculated alpha (the initial slope of the curve—a measure of light harvesting efficiency) and ETR_MAX_ (the asymptote of the curve—a measure of photosystem capacity to use absorbed light) [35].

Next, we removed pots and separated the original terminal shoot from the pot and trimmed the rhizome to 2.5 cm for nutrient uptake analysis using methods described by Terrados & Williams [36]. We placed the shoot into two compartment chambers with the belowground tissue incubated in unstirred artificial seawater spiked with ammonium (0.1 M), and the aboveground tissue incubated in artificial seawater spiked with nitrate (0.04 M). We held these chambers within larger, temperature-controlled plexiglas cylinders supplied with photosynthesis-saturating light and turbulent flow around the aboveground chambers to prevent mass transfer limitation [37]. We maintained water temperature at mean mesocosm temperature at the time of harvest, which increased as the summer progressed (acclimation 14°C; treatment: ambient 16°C and warm 21°C; recovery 17°C). We sampled water in the aboveground chamber before plants were added and at one hour intervals for 4 hours, and analyzed for nitrate concentration using a Lachat 8000 series flow injection auto analyzer. Following the trial, we separated plant tissue into above and belowground material, dried at 60° C for 48 h, and weighed it. Changes in nitrogen concentration were standardized by leaf biomass, and linear regression was used to estimate biomass specific uptake over time.

We estimated leaf growth, shoot production, and rhizome elongation in order to describe plant productivity. Since the number and size of shoots were initially standardized, we quantified the total number of shoots (minus the planted terminal shoot) and total rhizome length (minus the initial 3 cm), as well as dry biomass at the end of the time period as measures of production. Leaf elongation was estimated using the terminal shoot in each pot (the same shoot used to measure nutrient uptake), and new growth was calculated as leaf area and dry biomass produced using a needle prick marker [38]. We also used the terminal shoot to measure maximum leaf length, leaf width, number of leaves, and dry aboveground biomass per shoot.

### Statistical analysis

We first used a split plot mixed model ANOVA (Proc MIXED, SAS 9.1; SAS Institute, Cary, NC, USA) (Fixed factors: genotype and temperature; Random factors: mesocosm and interactions with mesocosm) to analyze data from the each of the time periods independently. Satterthwaite’s approximation of denominator degrees of freedom was used. This allowed us to assess whether there were initial differences in performance among mesocosms assigned to the different temperatures and to determine immediate effects of temperature during each time period. In order to better understand the effects of time and season, we analyzed the data from the control mesocosms across all time periods using a split plot mixed model ANOVA (Fixed factors: genotype, time; Random factors: mesocosm and interactions with mesocosm). Finally, we analyzed the effects of genotype, temperature (control vs. elevated), and time (treatment vs. recovery) using the same framework, allowing us to test whether genotype response to temperature varied.

Because analyses indicated a genotype-specific response to temperature, especially during the recovery phase, we investigated morphological or physiological correlates of these differences using a multiple linear regression with independent variables selected using the stepwise method (Proc REG, SAS 9.1). We estimated warming response as difference in number of new shoots between elevated plants at the end of the warming period and the end of the recovery period. We used morphological and physiological traits as independent variables to predict warming response. Traits were added and retained to the model using a threshold of p<0.15. In a separate analysis, we used the genotype specific phonological variation (baseline differences in shoot production between the acclimation period and the warming period (from control plants that were not influenced by elevated temperature)) as a dependent variable to explain genotype differences in warming response (estimated as above). Because controls were not directly paired with treatment, this analysis used the genotype means.

## Results

**Fig 1:**
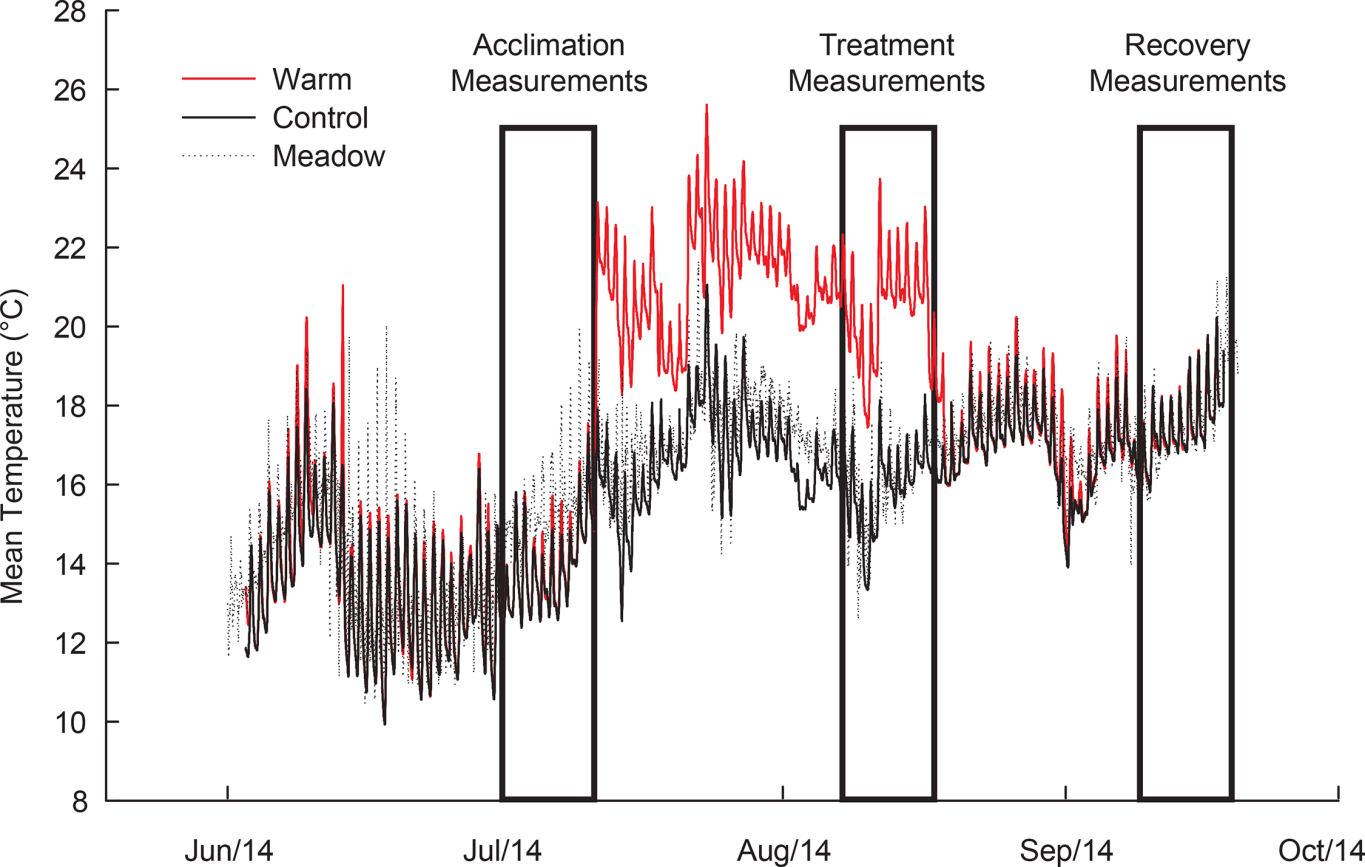
Experimental Temperature Manipulation. Solid lines represent the mean temperature in experimental mesocosms (red = elevated; black = control; n = 8 for each). The dotted line represents ambient temperature in a nearby eelgrass meadow. Boxes indicated the time periods when plant morphology, productivity, and physiology were analyzed.

During our 15 week duration experiment, mean temperature in controls increased naturally (seasonally) from 13.7°C during the acclimation phase to 17.1°C during the recovery phase. This is similar to what we observed in intertidal eelgrass meadows in the field during this period (Fig 1). During the warming period of the experiment, control mesocosms had a mean temperature of 16.5°C, while the warmed mesocosm were elevated 4.5°C with a mean of 20.9°C. Daily fluctuations in mesocosm temperatures were similar to those in the natural beds. Over the course of the experiment, water column nitrate values varied an order of magnitude between 2.0 and 20.0 μM, likely due to episodic upwelling events, but overall they did not vary between temperature treatments (data not shown; t = -0.84, p = 0.4). Daytime measurements of light and dissolved oxygen were always saturating. Over the entire experiment only ten of the 336 pots exhibited total plant mortality, and this limited mortality did not differ among temperatures, mesocosms, or genotypes.

**Fig 2:**
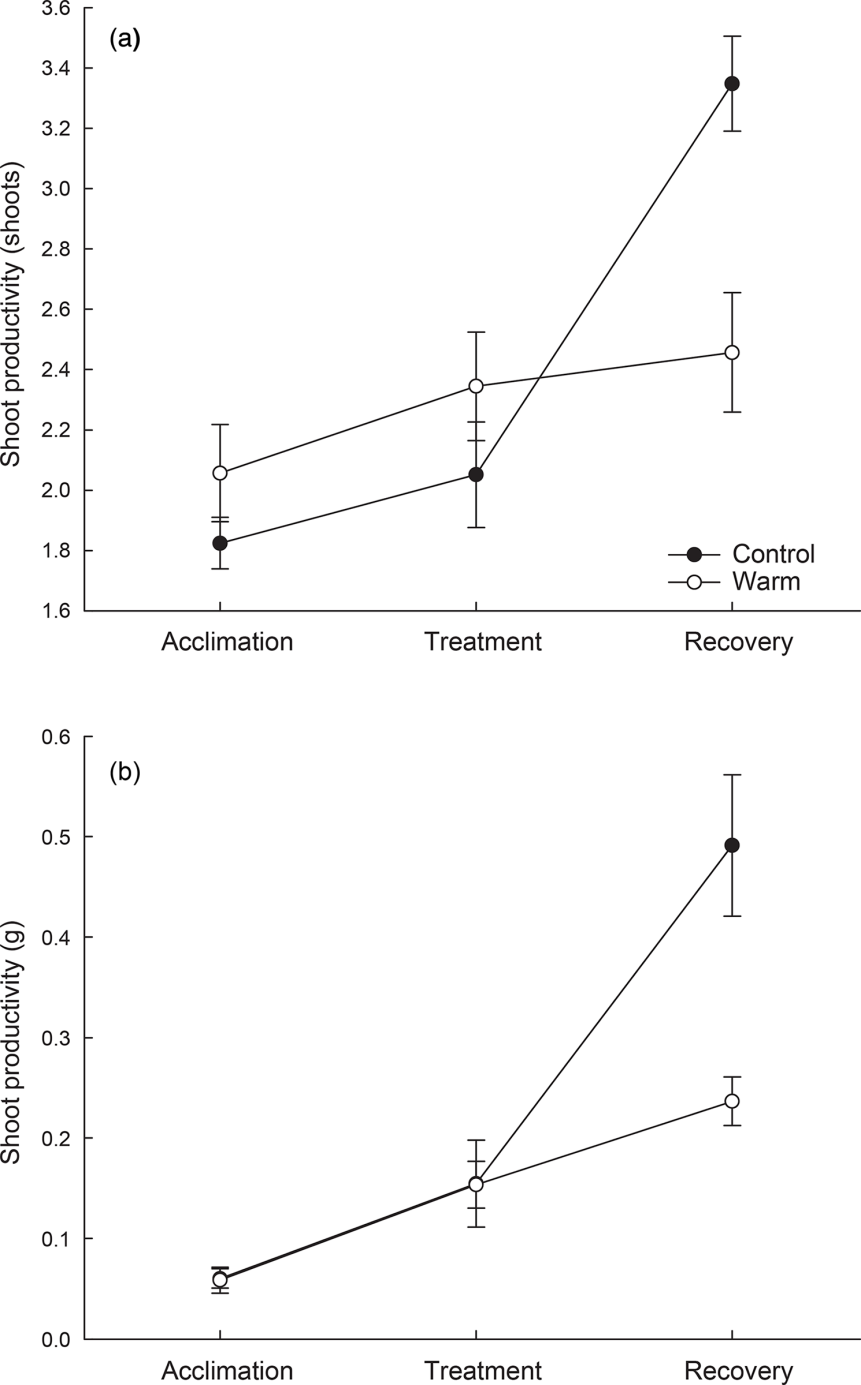
Mean cumulative shoot production by count (a) and biomass (b) over a 15 week experiment. During the acclimation and recovery period, growing conditions did not vary between treatments, but during the warming period, plants in the warm treatment were grown in temperatures elevated by 4.5°C. Means are of 7 genotypes (n = 8 per genotype), and error bars represent standard error.

This modest experimental increase in temperature had a lasting effect on new shoot production, measured by cumulative production of new shoots over the entire experiment. At the end of the acclimation period, genotypes varied in their new shoot production rates; however, there was no difference between mesocosms that were assigned to control vs. warming treatment (Table A in S1 File). At the end of the warming period, shoots under elevated temperatures had 17% greater shoot production than controls (Fig 2A, Table 1). There was no effect of elevated temperature on biomass production (Fig 2B), indicating a trend toward increased number of shoots that were smaller on average. The effect of elevated temperature reversed in direction and increased in magnitude during the recovery phase. Plants that had previously been more productive under elevated temperature had dramatic reductions in both shoot (27%) and biomass (50%) production after the end of the recovery phase (Fig 2).

**Table 1:** Results of the mixed model split plot ANOVA exploring the effects of genotype, time (acclimation, treatment, recovery), and interactions on new shoot production in plants in control mesocosms (never exposed to elevated temperature). Additional response variables (photosynthetic physiology, nutrient uptake and leaf growth shown in Table B in S2 File).

	New shoot production (mass)			New shoot production (count)		
	F	df	p	F	df	p
Genotype	2.14	50.94	2	<0.0001	52.04	2
Time	50.94	2	<0.0001	52.04	2	<0.0001
Genotype*Time	2.33	12	0.0093	0.98	12	0.47

**Fig 3:**
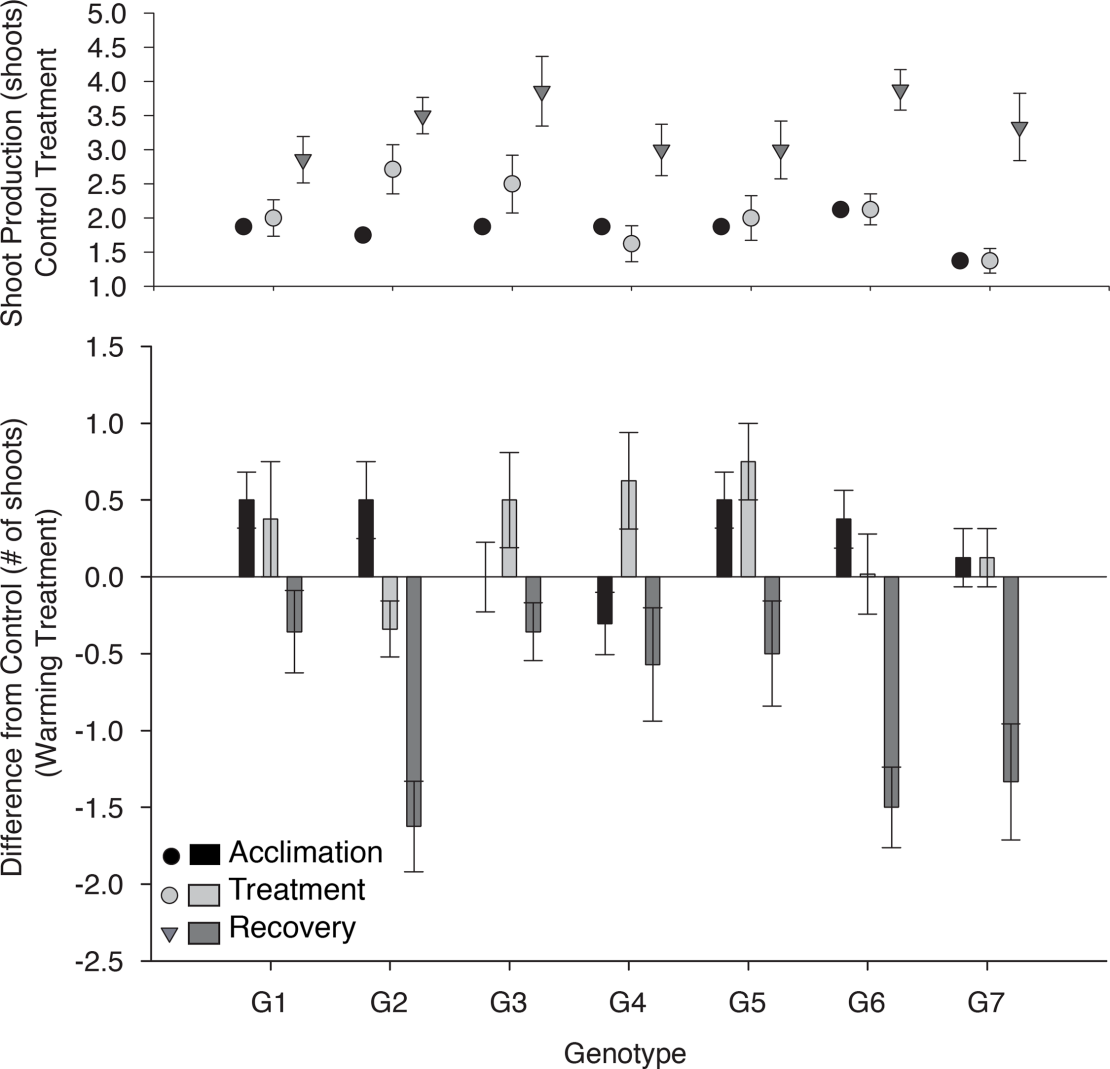
(a) Cumulative shoot production (counts) for 7 distinct *Zostera marina* genotypes over time. Dots are means of 8 replicates and error bars represent standard error. All plants were grown under ambient conditions and time periods were separated by 5 weeks. (b) An additional set of plants were grown under ambient conditions for 5 weeks (black bars), under temperature elevated by 4.5°C for 5 weeks (light grey bars), and again at ambient temperature for 5 weeks (dark grey bars). Bars represent the difference in shoot production from controls (always grown under ambient conditions), and error bars are standard error.

Individual genotypes varied in their rates of shoot production (Tables 1 and 2 Table A in File); however, those genotypic differences varied over time in control, and in elevated temperature treatments. Under control conditions, some genotypes initially grew quickly and leveled off, whereas others had an initial period of very slow growth followed fast growth later in the season (Fig 3, Genotype x Sampling Time Table 1). Across all experimental pots, mean density at the end of this experiment was 3.4 shoots per 80 cm^2^ pot, which is considerably lower than the maximum and mean field density of 900 and 500 shoots m^-2^ in Bodega Bay, respectively (= 7.5 and 4.0 shoots per 80 cm^2^) (Hughes & Stachowicz 2009), suggesting differences over time are a result of differing phenologies among genotypes rather than reaching carrying capacity (Fig 3A). In general, genotypes exposed to elevated temperature increased shoot production during the warming period and decreased shoot production during recovery, some genotypes were more tolerant of warming (genotype x temperature interaction, Table 2)) (Fig 3B).

**Table 2:** Results of the mixed model split plot ANOVA exploring the effects of genotype, temperature, time, and interactions on new shoot production. Results for additional response variables (plant physiology, morphology, and growth) are in Table C in S2 File. The warming treatment was a simulated 5 week event with temperature elevated by 4.5°C. There were 2 sampling time periods: immediately following the warming event and following a 5 week recovery when both treatments were exposed to ambient temperature.

	New shoot production (mass)			New shoot production (count)		
	F	df	p	F	df	p
Genotype	1.62	6	0.15	5.18	6	0.0001
Treatment	10.1	1	0.008	6.62	1	0.01
Time	41.71	1	<0.0001	35.59	1	<0.0001
Genotype* Treatment	0.88	6	0.5	2.17	6	0.05
Genotype* Time	2.63	6	0.02	1.35	6	0.24
Treatment* Time	15.3	1	0.0002	27.67	1	<0.0001
Genotype* Treatment* Time	1.7	6	0.13	0.31	6	0.9

Morphological traits, productivity, and chlorophyll fluorescence also varied by plant genotype (Tables A–C in S1 File). Many of those traits responded to warming in a matter similar to shoot production in that initially, plants generally responded positively to warming—greater number of newly produced shoots, longer rhizomes, higher dark adapted yield, and higher ETR_MAX_ (Figs A-L in S2 File). In most cases, the longer-term effect of warming was negative. During the recovery period, plants that had been previously warmed had fewer newly produced shoots, lower leaf elongation rates, shorter rhizomes, and were generally smaller (narrower leaves, reduced aboveground to belowground ratio) and took up nutrients at a lower rate. The dark adapted yield was also generally reduced compared to control plants; however, there was a genotype specific response (significant genotype x temperature effect) (Table C in S1 File). Dark adapted yield values remained within the optimal range for seagrass (0.7–0.8) [39,40,41] (Fig H in S2 File).

**Fig 4:**
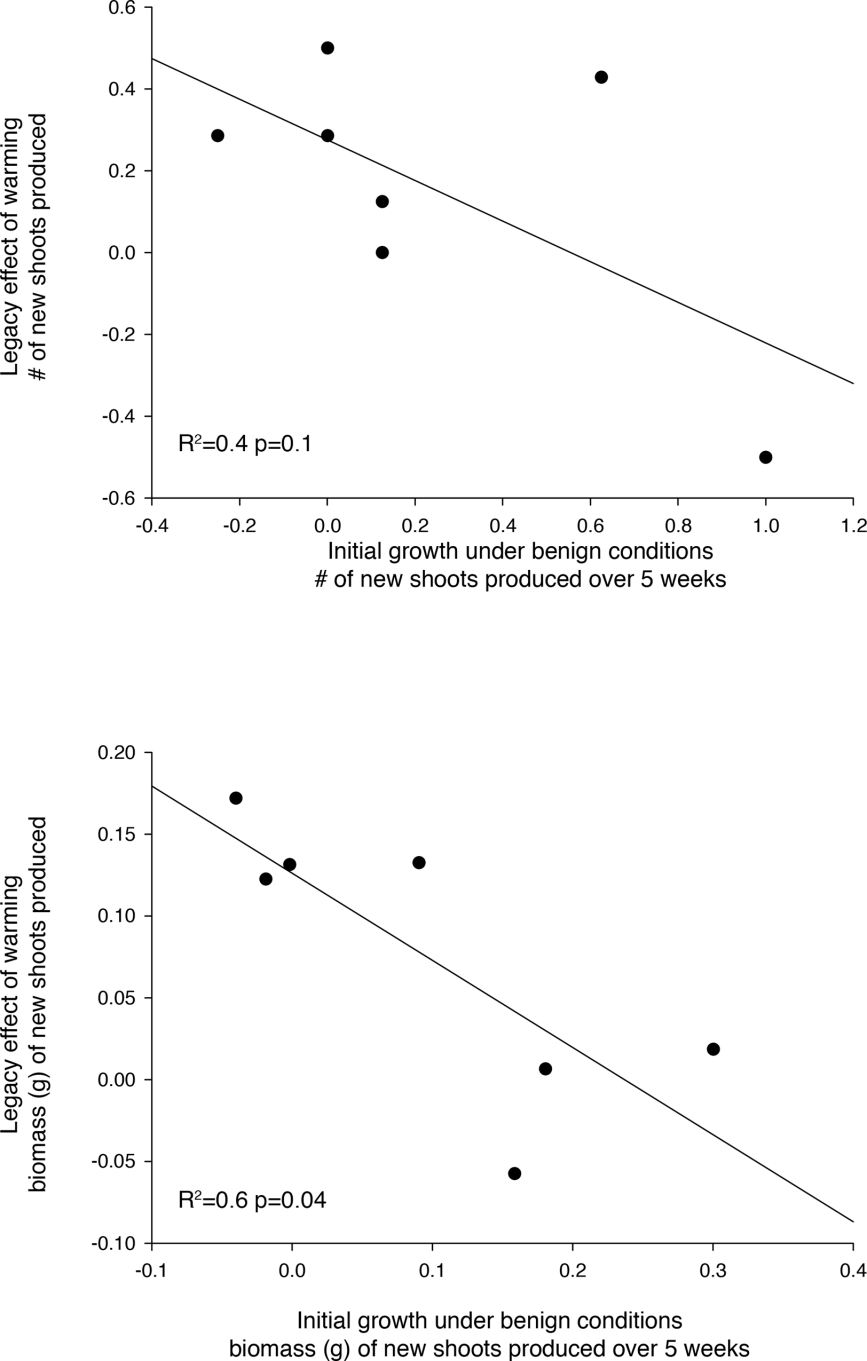
Initial growth (between the acclimation period and the warming period from control plants that were not influenced by elevated temperature) is negatively correlated with growth during the recovery phase of previously warmed plants, as measured by the difference in shoot counts (m = -0.5) (a) and by biomass (m = -0.5) (b). Each point is the mean for a particular genotype.

None of the measured traits explained more than 16 percent of the variability in the shoot production (biomass or count) response to warming (Table 3). However, initial production rate of a genotype in the control treatment was negatively correlated with the reduction in shoot production that occurred during the recovery phase (biomass after the recovery period minus biomass at end of warming period). Genotypes that grew quickly initially suffered a greater reduction in shoot production and aboveground biomass during the recovery from warming (Mass: R^2^ = 0.6 p = 0.04; Count: R^2^ = 0.4 p = 0.1) (Fig 4). Analyzing the trait data in an analogous manner (means of genotypes) resulted in no significant correlations with warming response.

**Table 3:** Multiple Step-wise Regression Approach used to assess the relationship between traits of genotypes measured at the beginning of the experiment (acclimation period) and change in shoot production due to elevated temperature. We estimated new shoot production two ways: (a) difference in number of new shoots between elevated and the mean of control genotypes at the end of the recovery period and (b) difference in number of new shoots produced under elevated temperature at the end of the warming period vs. the end of the recovery period.

Response Variable	Explanatory Variable	Partial R^2^	F	p
Biomass	Number of Leaves	0.16	5.5	0.02
Counts	Nitrate uptake	0.14	0.49	0.04

## Discussion

In this study, we documented genotype-specific responses to modest experimental warming likely to be experienced in the near future. There was a modest increase in eelgrass shoot production, but not biomass accumulation when exposed to warmed water for short duration (up to 5 weeks). When temperatures returned to ambient, however, shoot size, productivity, and rates of nutrient uptake decreased for at least 5 weeks. While all genotypes exhibited this pattern, some genotypes had a greater decrease in performance than others (significant G X E effect). Genotypes that grew the most rapidly under control conditions suffered the largest negative consequences of warming. This suggests that warming or other stress events of sufficient frequency could both maintain diversity in the population and lead to enhanced production in diverse stands.

Like studies of other species with large geographical ranges (e.g.[42]), our results suggest that eelgrass many have a different local thermal tolerance than the total climate envelope for the species as a whole. In this species, at high temperatures (30–35°C), a negative carbon balance can occur as respiration outpaces increased photosynthesis as temperature increases [43,44] and production of heat shock proteins associated with declining production increases [45,46,47]. However, Bodega Bay eelgrass populations are sensitive to short-term warming even though they are growing in roughly the center of its eastern Pacific latitudinal distribution, in northern California waters where ambient temperatures at the field site are below optimum [48,49]. The experimental warming of 4.5°C to a maximum of 25°C never exceeded values previously established as stressful (28–30°C) for *Z*. *marina* [50,51,52], and in fact the warming treatment mean of 21°C was very close to the optimum temperature for eelgrass as a species [47,48]. While the threshold for eelgrass biomass decline can be lower if there are additional environmental stressors (i.e. reduced light) [7,53], these were not present in our experiment. (Light was saturating, and water column nitrate values were sufficiently high.) Furthermore, although warming initially had no negative effect on eelgrass production, negative legacy effects of warming manifested weeks after plants had been returned to ambient conditions. The legacy negative effect indicates that, despite what might initially appear as a positive effect of warming in eelgrass well within its physiological temperature envelope, eelgrass production can be negatively affected by warming upon return to mean ambient conditions.

The delay in response to heat stress is likely a result of the ability of seagrasses to use carbohydrate stores in the rhizomes to maintain (or even increase) growth for short periods of time despite stresses [54,55]. Salo *et al*. [26], found that under light stress, the use and restocking of these stores varied by genotype. Differential use of storage could explain the interactive effects of genotype and warming treatment found here, but we found no evidence that belowground biomass was reduced in response to shoot growth. Structures storing resources for later use are present in species across a broad taxonomic range store (i.e. rhizomes in salt marsh grasses [56], fern rhizomes [57], fat stores in whales [58], water storage in cacti [59]) suggesting that this temporal mismatch may be common in nature. Therefore, frequent and long-term monitoring is likely very important in interpreting the impacts of stress events in natural systems.

All genotypes responded negatively to warming during the recovery phase, but some were more tolerant of heat stress than others. This provides a mechanism for increased stress resistance observed in meadows that are genetically diverse. For either selection or niche partitioning resulting in complementarity to occur, plants must differ in their functional traits in ways that allow them to exploit different resource bases or environments [13]. Trait variation among genotypes in common garden is well known for both eelgrass [24,25] as well as other plants [60,61] and across many species. Trait differences often depend on environmental conditions [62,63] and in this case, the relative impact of environment is genotype specific [64,65,66,67]. Here we show a basis for how trait variation can mediate responses to environmental variation. Specifically, there appears to be a tradeoff between rapid growth and maintenance of growth in elevated temperature, such that genotypes with early-growing phenologies or that are favored in ambient temperature conditions are less tolerant of high temperatures than those that grow more later in the season. Thus, having a mix of genotypes could stabilize production and promote co-existence of clones in field plots over the course of a season as baseline temperature changes or across years that vary in temperature.

Biodiversity at any level can enhance ecosystem productivity and stability by increasing trait and thus functional diversity [68,69], but separating species or genotypes into functional groups is difficult as it relies on a good understanding of trait diversity as well as the ability to define relevant traits [70]. Our results indicate that snapshot measures of trait diversity may not fully describe genotypic differences that are relevant to plant response to environmental change. For example, at 5 weeks, genotypes 6 and 7 had low recruitment and genotype 2 had high recruitment; however at 15 weeks, all three genotypes had high and statistically similar numbers of recruited shoots. Shoot density in pots was well within that observed in the field, so these differences do not appear to be the result of genotypes reaching carrying capacity at different rates; indeed, shoot density was higher in ambient than elevated temperature mesocosms at the end of the experiment, also suggesting a net cost to warming.

The phenological variation in plant traits may aid in the maintenance of population genetic diversity. Bodega Harbor eelgrass meadows are very diverse in terms of both genotypic and allelic richness relative to other populatons [71,72], and these data are consistent with the idea that relatively benign but variable conditions may promote maintenance of this diversity. When gaps are created by disturbance, individuals with high initial growth and clonal reproduction rates are likely to dominate; however, our data suggest that these individuals are likely to be poor competitors under stressful (i.e. warm) conditions. Therefore, episodic stressful temperatures (or alterative stressors such as low light) may shift competitive advantage and allow for greater co-existence and increased genetic diversity. In fact, the high intertidal zone in Bodega Harbor, where air exposure, temperature, and environmental variability is greatest, shows a higher genotypic diversity than the subtidal zone [73]. Likewise, in a repeat sampling of eelgrass meadows in Brittany France, Becheler *et al*. [74] reported that over a 3-year period, seasonal reductions in shoot density of dominant clones facilitated transient colonization by new recruits. The role of stressors and environmental variability in the maintenance of eelgrass meadow diversity sets up a potential positive feedback since genetically diverse assemblages show more resistance and resilience to further disturbances. Analogously, phenologies (i.e. bud burst and flowering) change with increased temperature and precipitation in a species-specific manner altering assemblages and competition in a many habitats ranging from grasslands to forests [75,76,77].

The diversity and temporal variability of genotype specific traits support the idea that restorations aimed at counteracting or reducing climate change impacts should consider the genotype specific traits when selecting donor material [18]. However, the selection of genotypes that will perform well is challenging. When selecting from stock cultured in the laboratory, our data demonstrate that multiple measures of plant traits over time are needed to best understand how genotypes will respond to stress, and it is unknown whether genotypes tolerant of warming will also be more resistant to other stresses such as nutrient loading or light reduction. The contrasting approach—selecting material from source locations with conditions that match the restoration location—is also not straightforward. The variation in temperature response found among genotypes from within Bodega Bay is similar or even greater than the differences found when using plants from different climatic regions [44,45], which underscores the importance of a full understanding of trait variation and the selection acting upon traits on large and small spatial scales when selecting stock [78].

## Supporting Information

S1 File Additional statistical tables A–C. (PDF)

S2 File Morphology, physiological, and productivity figures from each time period (acclimation, treatment, and recovery) A–L. (PDF)
