# Supplementary material for: Response of a Habitat-Forming Marine Plant to a Simulated Warming Event Is Delayed, Genotype Specific, and Varies with Phenology
Source: PLoS One. 2016 Jun 3;11(6):e0154532. doi: 10.1371/journal.pone.0154532 (PMC4892549; doi:10.1371/journal.pone.0154532)
Supplement: S1 File — (PDF) [file pone.0154532.s001.pdf]

Response of a Habitat-Forming Marine Plant to a Simulated Warming Event is Delayed, Genotype Specific, and Varies with Phenology

Laura K. Reynolds[1*]

Katherine DuBois[1,2]

Jessica M. Abbott[1]

Susan L. Williams[1,2]

John J. Stachowicz[1]

[1] Department of Evolution and Ecology; University of California Davis; Davis, CA 95616

[2] Bodega Marine Lab; University of California Davis; Bodega Bay, CA 94923

[*] Corresponding author: lkreynolds@ucdavis.edu

Supplement S1. Additional Statistical Tables

Supporting information

Table A in File S1. ANOVA table for each period individually. Table showing the results of the mixed model split plot ANOVAs exploring the effects of treatment and interactions with treatment on plant physiology, morphology, and growth plants in after an acclimation time period where all plants were treated identically, after a 5 week warming event where half of the plants were exposed to water temperatures that were elevated 4.5°C above ambient, and a subsequent 5 week recovery period where all plans were exposed to ambient temperatures.

Acclimation Time Period												
Physiology	Dark adapted yield			Alpha			ETR Max			NO3 uptake		
	F	df	p	F	df	p	F	df	p	F	df	p
Genotype	0.98	6	0.44	0.31	6	0.93	1.33	6	0.27	1.45	6	0.21
Treatment	0.16	1	0.69	0	1	0.98	0.14	1	0.71	0.17	1	0.68
Genotype* Treatment	1.93	6	0.087	3.53	6	0.0064	0.72	6	0.6	0.42	6	0.86
Morphology	Leaf Length			Leaf Width			# of leaves			Above:below		
	F	df	p	F	df	p	F	df	p	F	df	p
Genotype	3.05	6	0.0095	2.04	6	0.07	2.09	6	0.06	1.36	6	0.23
Treatment	0.07	1	0.79	0.47	1	0.5	0.01	1	0.93	0.33	1	0.57
Genotype* Treatment	1.46	6	0.21	1.09	6	0.37	0.93	1.6	6	0.74	6	0.62
Productivity	Leaf Productivity (g)			Leaf Productivity (cm)			Rhizome					
	F	df	p	F	df	p	F	df	p			
Genotype	3.9	6	0.0016	1.07	6	0.38	7.62	6	<0.001			
Treatment	2.71	1	0.1	2.63	1	0.12	3.21	1	0.09			

Genotype* Treatment	0.2	6	0.98	1.09	6	0.34	1.04	6	0.4			
New Shoot production	Shoot Productivity (g)			Shoot Productivity (cm)								
	F	df	p	F	df	p						
Genotype	2.41	6	0.033	4.3	6	0.0008						
Treatment	0.01	1	0.91	3.41	1	0.09						
Genotype* Treatment	0.64	6	0.7	1.35	6	0.24						

Treatment Time Period												
Physiology	Dark adapted yield			Alpha			ETR Max			NO3 uptake		
	F	df	p	F	df	p	F	df	p	F	df	p
Genotype	2.2	6	0.05	0.77	6	0.59	0.89	6	0.51	0.96	6	0.45
Treatment	2.79	1	0.098	2.12	1	0.15	26.73	1	<0.001	2.57	1	0.13
Genotype* Treatment	1.47	6	0.19	1.65	6	0.15	1.76	6	0.12	1.61	6	0.16
Morphology	Leaf Length			Leaf Width			# of leaves			Above:below		
	F	df	p	F	df	p	F	df	p	F	df	p
Genotype	5.41	6	0.0001	2.04	6	0.07	2.81	6	0.02	1.65	6	0.14
Treatment	2.89	1	0.11	0.35	1	0.57	4.75	1	0.047	0.23	1	0.63
Genotype* Treatment	0.49	6	0.81	0.47	6	0.83	0.047	0.38	6	1.3	6	0.26
Productivity	Leaf Productivity (g)			Leaf Productivity (cm)			Rhizome					
	F	df	p	F	df	p	F	df	p			
Genotype	1.08	6	0.34	3.15	6	0.0081	15.44	6	<0.0001			
Treatment	0.17	1	0.69	0.03	1	0.87	4.19	1	0.04			
Genotype*	0.54	6	0.77	1.09	6	0.38	0.57	6	0.74			

Treatment												
New Shoot production	Shoot Productivity (g)			Shoot Productivity (cm)								
	F	df	p	F	df	p						
Genotype	2.83	6	0.014	4.43	6	0.0005						
Treatment	0	1	0.99	3.6	1	0.051						
Genotype* Treatment	1.01	6	0.42	0.86	6	0.53						

Recovery Time Period												
Physiology	Dark adapted yield			Alpha			ETR Max			NO3 uptake		
	F	df	p	F	df	p	F	df	p	F	df	p
Genotype	1.53	6	0.18	1.4	6	0.22	2.67	6	0.022	3.6	6	0.0042
Treatment	1.06	1	0.32	1.59	1	0.21	0.25	1	0.62	24.49	1	0.0006
Genotype* Treatment	2.46	6	0.032	2.5	6	0.029	1.54	6	0.18	0.77	6	0.59
Morphology	Leaf Length			Leaf Width			# of leaves			Above:below		
	F	df	p	F	df	p	F	df	p	F	df	p
Genotype	3.51	6	0.0042	3.75	6	0.0024	1.19	6	0.31	3.9	6	0.0016
Treatment	3.84	1	0.069	12.92	1	0.0005	0.02	1	0.88	4.77	1	0.031
Genotype* Treatment	0.95	6	0.46	1.52	6	0.18	0.88	1.85	6	0.56	6	0.76
Productivity	Leaf Productivity (g)			Leaf Productivity (cm)			Rhizome					
	F	df	p	F	df	p	F	df	p			
Genotype	3.08	6	0.0097	2.92	6	0.01	9.88	6	<0.0001			
Treatment	1.49	1	0.0042	30.77	1	<0.0001	4.7	1	0.049			
Genotype* Treatment	0.68	6	0.67	0.49	6	0.81	1.23	6	0.3			

New Shoot production	Shoot Productivity (g)			Shoot Productivity (cm)								
	F	df	p	F	df	p						
Genotype	1.78	6	0.11	2.72	6	0.018						
Treatment	18.43	1	<0.0001	26.28	1	<0.0001						
Genotype* Treatment	1.33	6	0.25	1.62	6	0.15						

Table B in File S1. ANOVA table for control treatment alone. Table showing the results of the mixed model split plot ANOVA exploring the effects of genotype, time, and interactions on plant physiology, morphology, and growth. Temperature was not manipulated for plants used in this dataset. There were three sampling periods separated by 5 weeks.

Physiology	Dark adapted yield			Alpha			ETR Max			NO3 uptake		
	F	df	p	F	df	p	F	df	p	F	df	p
Genotype	1.31	6	0.25	1.17	6	0.32	0.065	6	0.68	0.95	6	0.47
Time	15.24	2	<0.0001	5.12	2	0.008	18.68	2	<0.0001	30.42	2	<0.0001
Genotype* Time	1.97	12	0.032	1.66	12	0.08	1.25	12	0.26	2.16	12	0.023
Morphology	Leaf Length			Leaf Width			# of leaves			Above:below		
	F	df	p	F	df	p	F	df	p	F	df	p
Genotype	4.79	6	0.0002	1.9	6	0.08	2.74	6	0.024	1.66	6	0.13
Time	66.71	2	<0.0001	9.3	2	0.0002	0.11	2	0.89	4.37	2	0.01
Genotype* Time	1.2	12	0.29	0.53	12	0.89	0.89	0.98	12	0.55	12	0.88
Productivity	Leaf Productivity (g)			Leaf Productivity (cm)			Rhizome					
	F	df	p	F	df	p	F	df	p			
Genotype	1.25	6	0.28	3.34	6	0.0043	13.64	6	<0.0001			
Time	40.87	2	<0.0001	24.49	2	<0.0001	146.93	2	<0.0001			
Genotype* Time	0.77	12	0.69	1	12	0.45	3	12	0.0013			

Table A in File S1. ANOVA table. Table showing the results of the mixed model split plot ANOVA exploring the effects of genotype, warming treatment, time, and interactions on plant physiology, morphology, and growth. The warming treatment was a simulated 5 week event with temperature elevated by 5 degrees. There were 2 sampling time periods: immediately following the warming event and following a 5 week recovery when both treatments were exposed to ambient temperatures.

Physiology	Dark adapted yield			Alpha			ETR Max			NO3 uptake		
	F	df	p	F	df	p	F	df	p	F	df	p
Genotype	2	6	0.068	1.67	6	0.14	2.42	6	0.029	0.59	6	0.73
Treatment	0.31	1	0.57	3.42	1	0.067	0.52	1	0.48	0.16	1	0.69
Time	27	1	0.6	4.44	1	0.037	0.01	1	0.91	74.44	1	<0.0001
Genotype* Treatment	2.57	6	0.02	1.02	6	0.41	0.82	6	0.56	1.5	6	0.18
Genotype* Time	1.35	6	0.24	1.35	1.35	1.35	1.67	6	0.13	1.45	6	0.2
Treatment* Time	4.86	1	0.028	0.1	1	0.74	11.46	1	0.0009	1.35	1	0.0011
Genotype* Treatment* Time	0.37	6	0.899	2.99	6	0.0087	1.87	6	0.089	1.35	6	0.19
Morphology	Leaf Length			Leaf Width			# of leaves			Above:below		
	F	df	p	F	df	p	F	df	p	F	df	p
Genotype	7.75	6	<0.0001	2.99	6	0.0086	1.84	6	0.094	5.11	6	<0.0001
Treatment	0.01	1	0.92	2.6	1	0.13	4.29	1	0.039	2.1	1	0.17
Time	2.03	1	0.16	6.93	1	0.0093	0.039	2.7	1	10.72	1	0.0013
Genotype* Treatment	0.89	6	0.51	0.86	6	0.52	2.7	1	0.1	0.7	6	0.65
Genotype* Time	1.02	6	0.41	1.82	6	0.098	0.38	1.89	6	0.7	6	0.65
Treatment*	1.02	6	0.41	7.45	1	0.0071	3.45	1	0.065	0.75	1	0.38

Time												
Genotype* Treatment* Time	0.048	6	0.82	0.84	6	0.54	1.41	6	0.21	1.59	6	0.15
Productivity	Leaf Productivity (g)			Leaf Productivity (cm)			Rhizome					
	F	df	p	F	df	p	F	df	p			
Genotype	2.02	6	0.06	4	6	0.0009	19.64	6	<0.0001			
Treatment	7.49	1	0.01	11.52	1	0.004	1.85	1	0.19			
Time	1.4	1	0.2	13.38	1	0.0003	103.4	1	<0.0001			
Genotype* Treatment	0.29	6	0.9	0.87	6	0.5	1.4	6	0.22			
Genotype* Time	1.08	6	0.38	1.31	6	0.25	0.74	6	0.62			
Treatment* Time	12.91	1	0.0004	12.99	1	0.0004	12.55	1	0.0006			
Genotype* Treatment* Time	0.73	6	0.63	0.76	1	0.61	0.53	6	0.8			
