# Supplementary material for: Response of a Habitat-Forming Marine Plant to a Simulated Warming Event Is Delayed, Genotype Specific, and Varies with Phenology
Source: PLoS One. 2016 Jun 3;11(6):e0154532. doi: 10.1371/journal.pone.0154532 (PMC4892549; doi:10.1371/journal.pone.0154532)
Supplement: S2 File — (PDF) [file pone.0154532.s002.pdf]

Response of a Habitat-Forming Marine Plant to a Simulated Warming Event is Delayed, Genotype Specific, and Varies with Phenology

Laura K. Reynolds[1*]

Katherine DuBois[1,2]

Jessica M. Abbott[1]

Susan L. Williams[1,2]

John J. Stachowicz[1]

[1] Department of Evolution and Ecology; University of California Davis; Davis, CA 95616

[2] Bodega Marine Lab; University of California Davis; Bodega Bay, CA 94923

[*] Corresponding author: lkreynolds@ucdavis.edu

S2 File. The following figures depict changes in physiological, morphological, and growth traits with plant genotype and warming treatment. In the top panel (a), dots are means of 8 replicates and error bars represent standard error. All plants were grown under ambient conditions and time periods were separated by 5 weeks. In the bottom panel (b), An additional set of plants were grown under ambient conditions for 5 weeks (black bars), under temperature elevated by 4.5°C for 5 weeks (light grey bars), and again at ambient temperature for 5 weeks (dark grey bars). Bars represent the difference from controls (always grown under ambient conditions), and error bars are standard error.

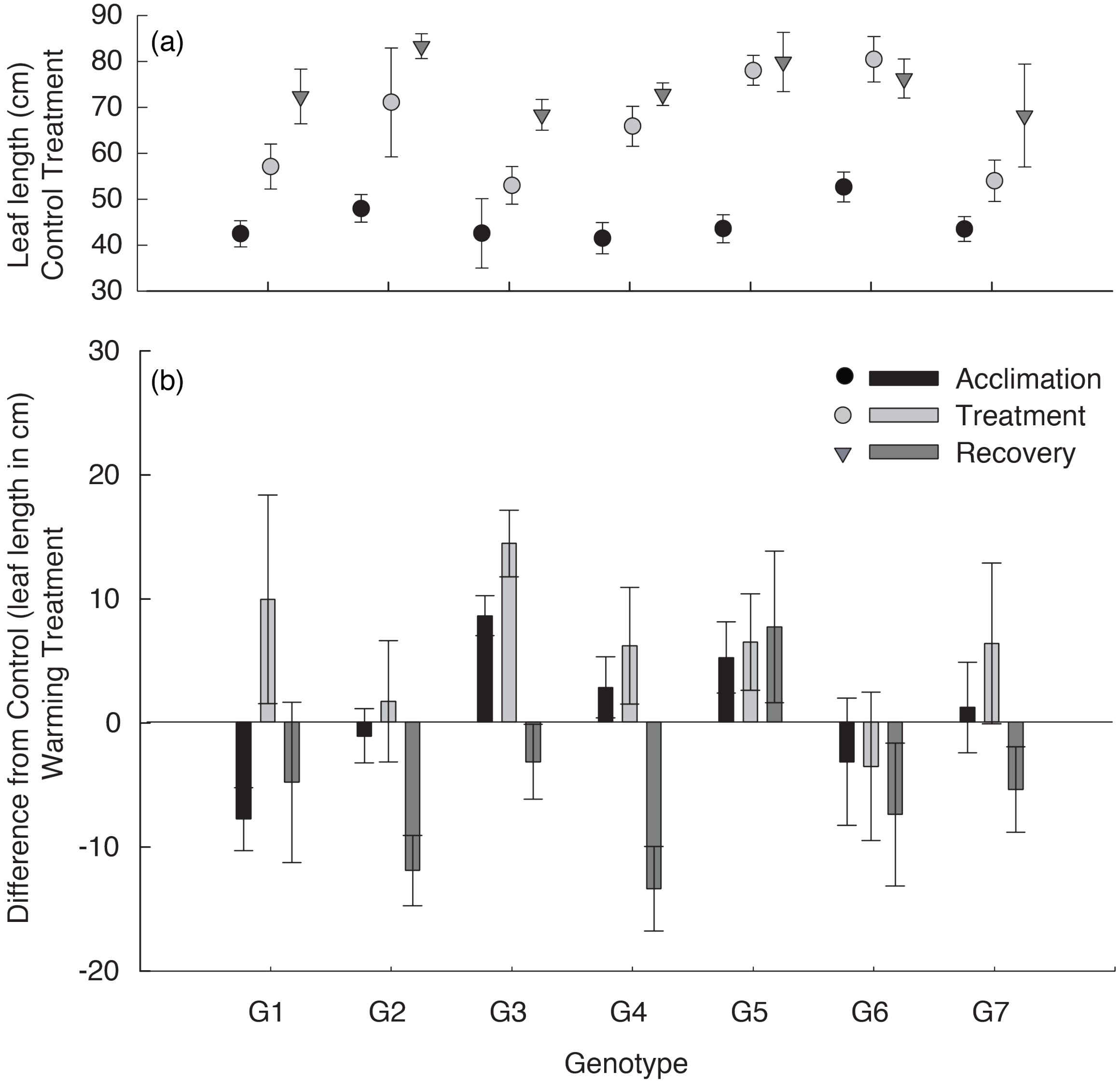

Figure A in S2. Terminal shoot leaf length for 7 distinct *Zostera marina* genotypes over time.

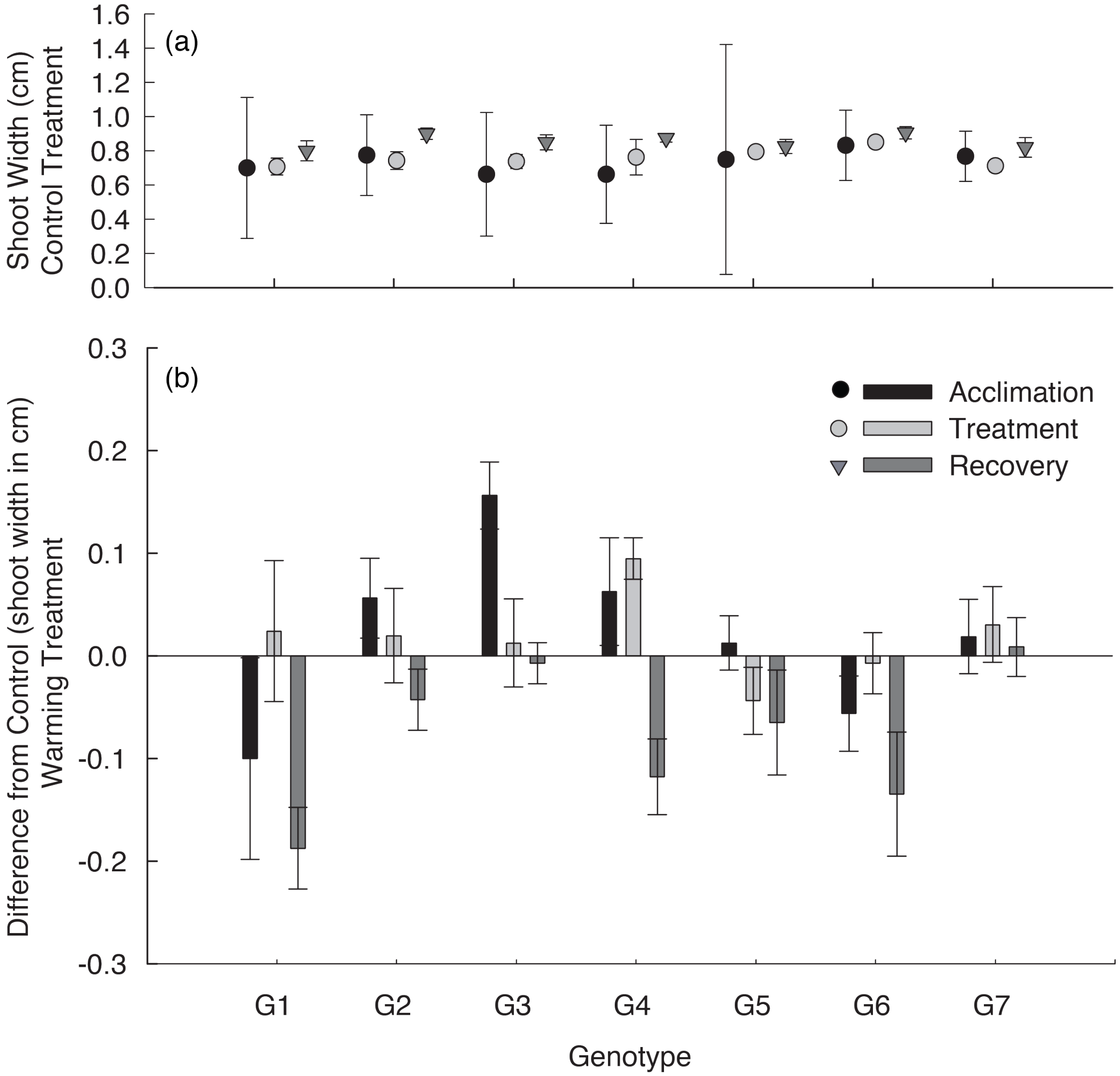

Figure B in S2. (a) Terminal shoot width length for 7 distinct *Zostera marina* genotypes over time.

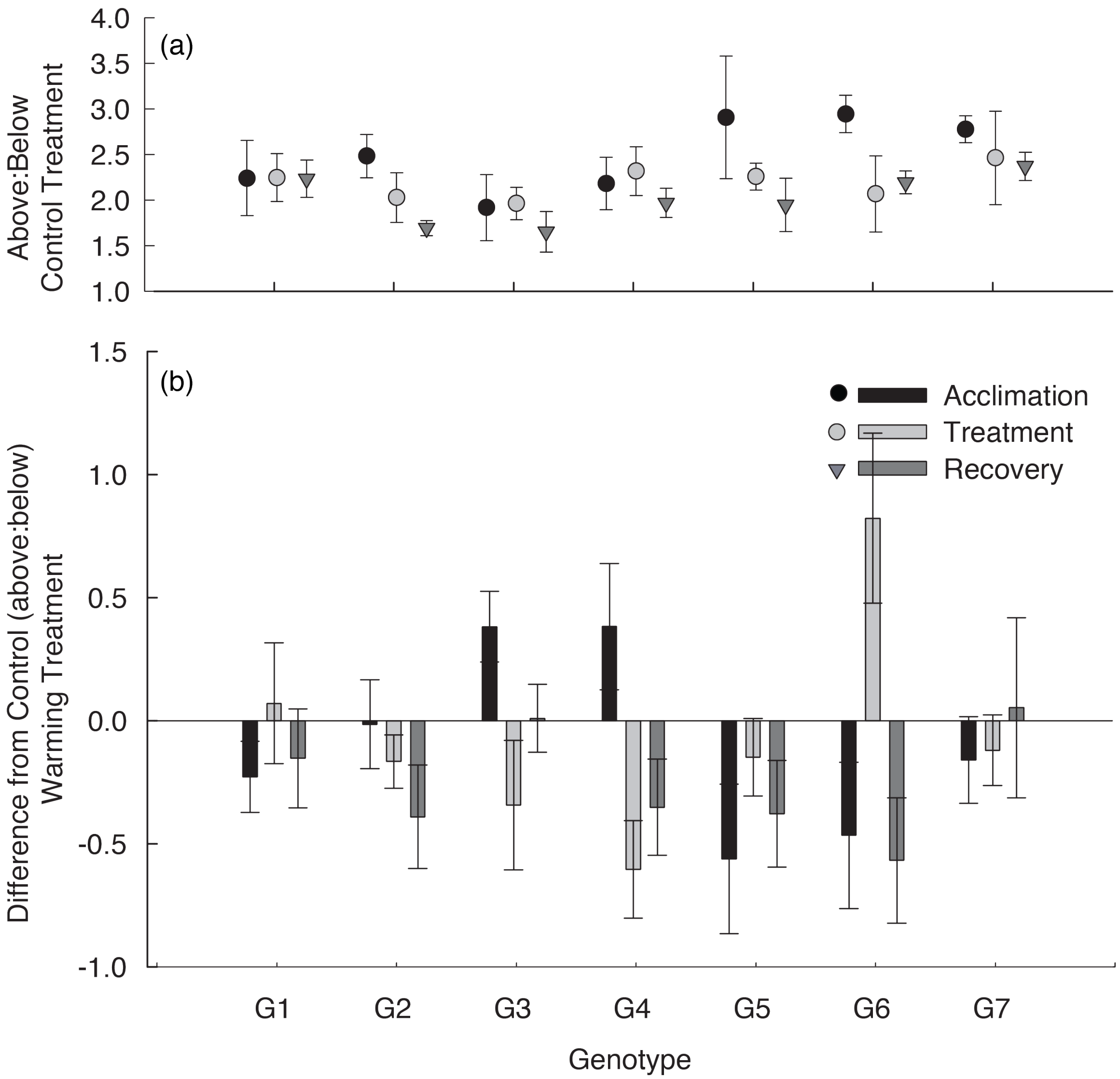

Figure C in S2. Above to below ground biomass ratio for 7 distinct *Zostera marina* genotypes over time.

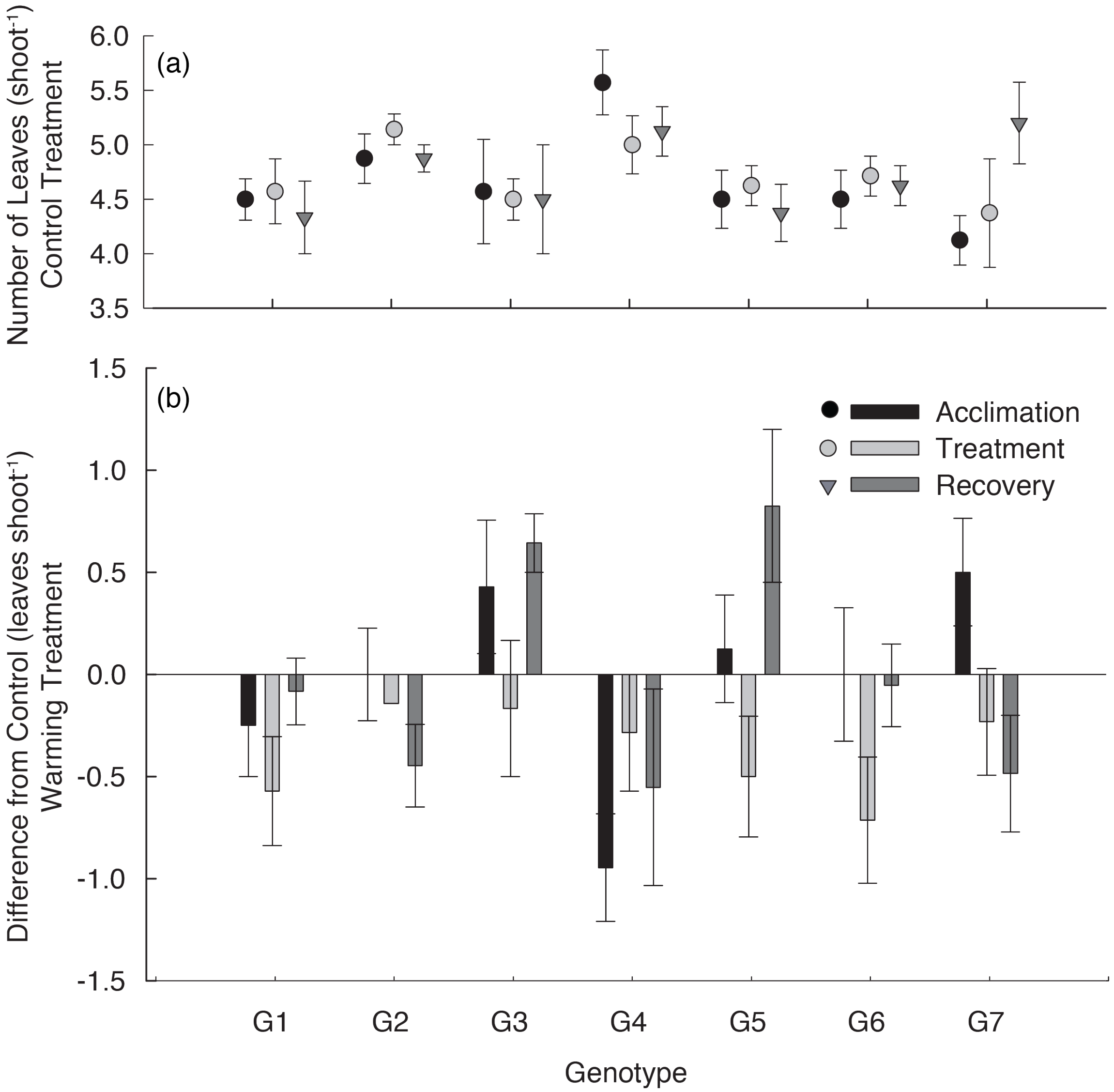

Figure D in S2. Terminal shoot leaf count for 7 distinct *Zostera marina* genotypes over time.

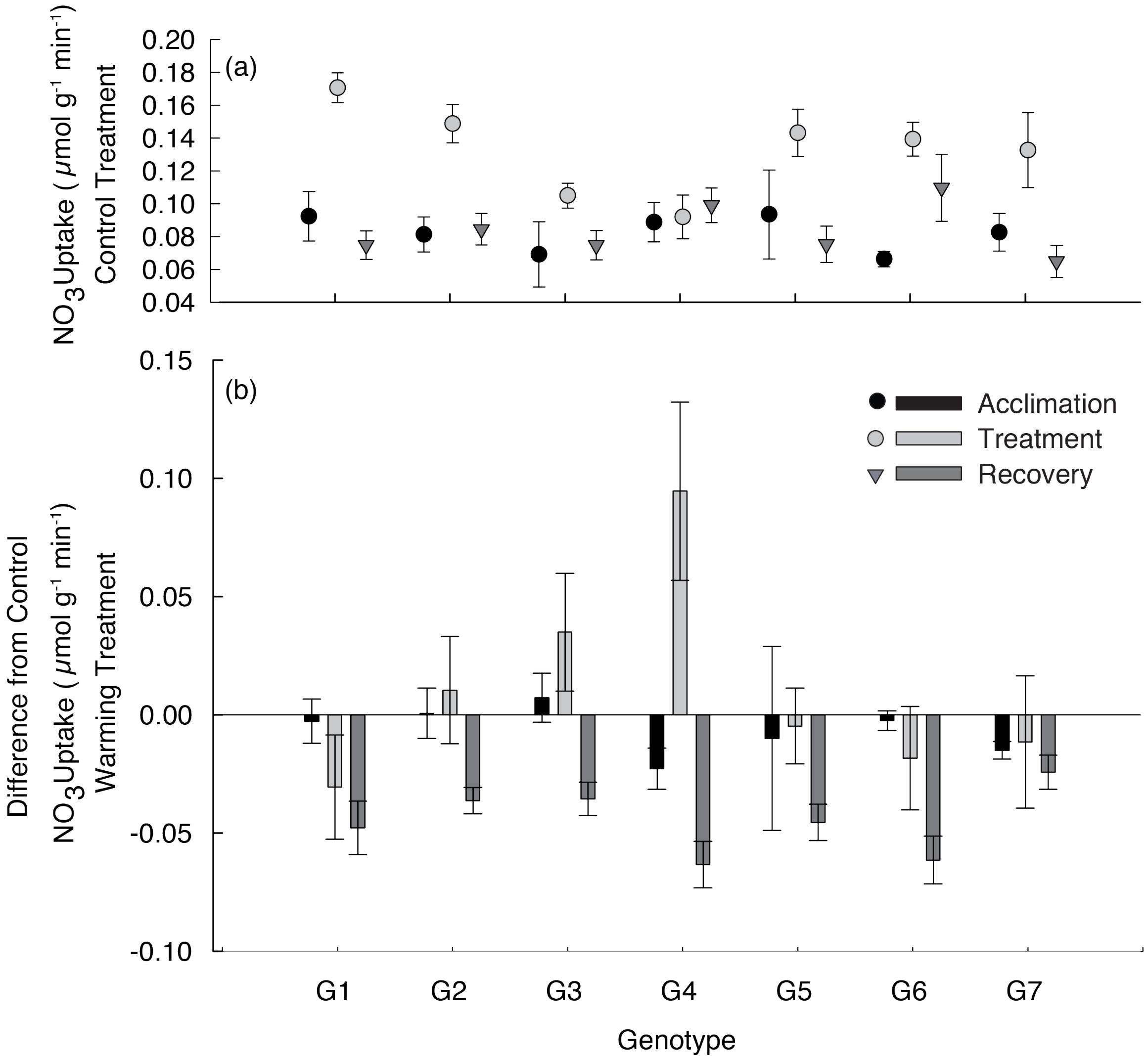

Figure E in S2. Terminal shoot nitrate uptake rate for 7 distinct *Zostera marina* genotypes over time.

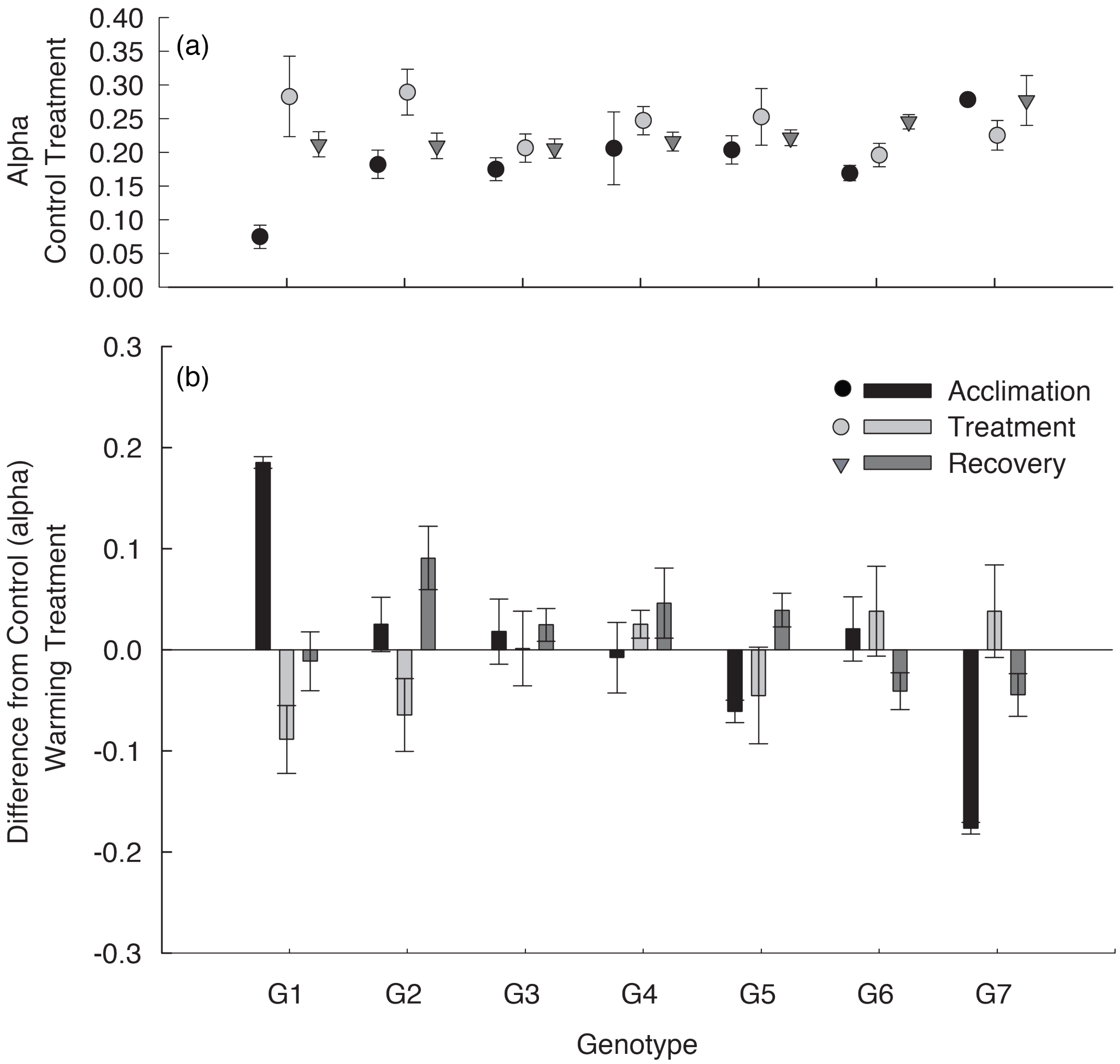

Figure F in S2. Alpha (the initial slope of the curve—a measure of light harvesting efficiency by Pulse Amplitude Modulation) for 7 distinct *Zostera marina* genotypes over time.

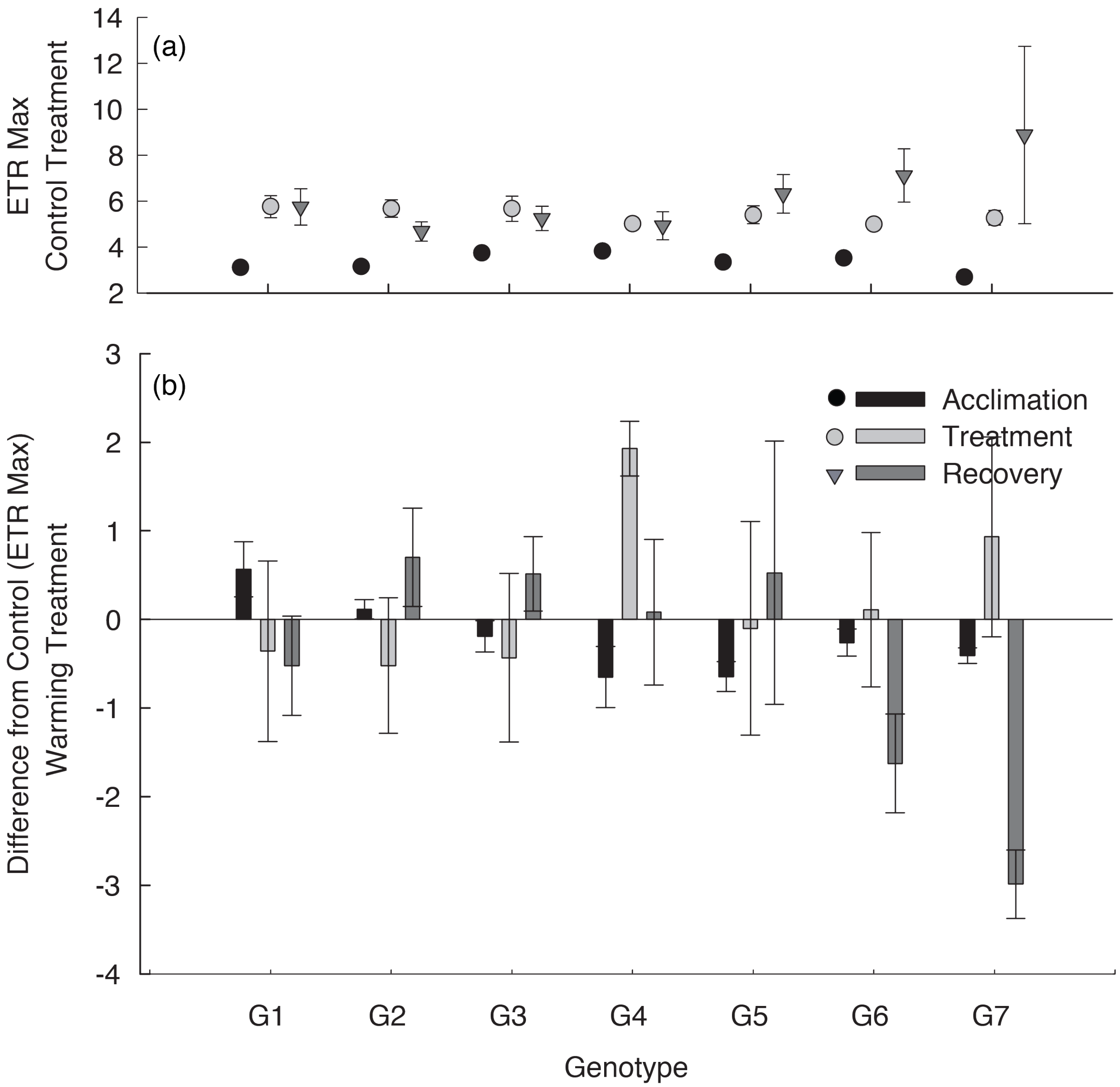

Figure G in S2. ETR_{MAX} (the asymptote of the curve—a measure of photosystem capacity to use absorbed light by Pulse Amplitude Modulation) for 7 distinct *Zostera marina* genotypes over time.

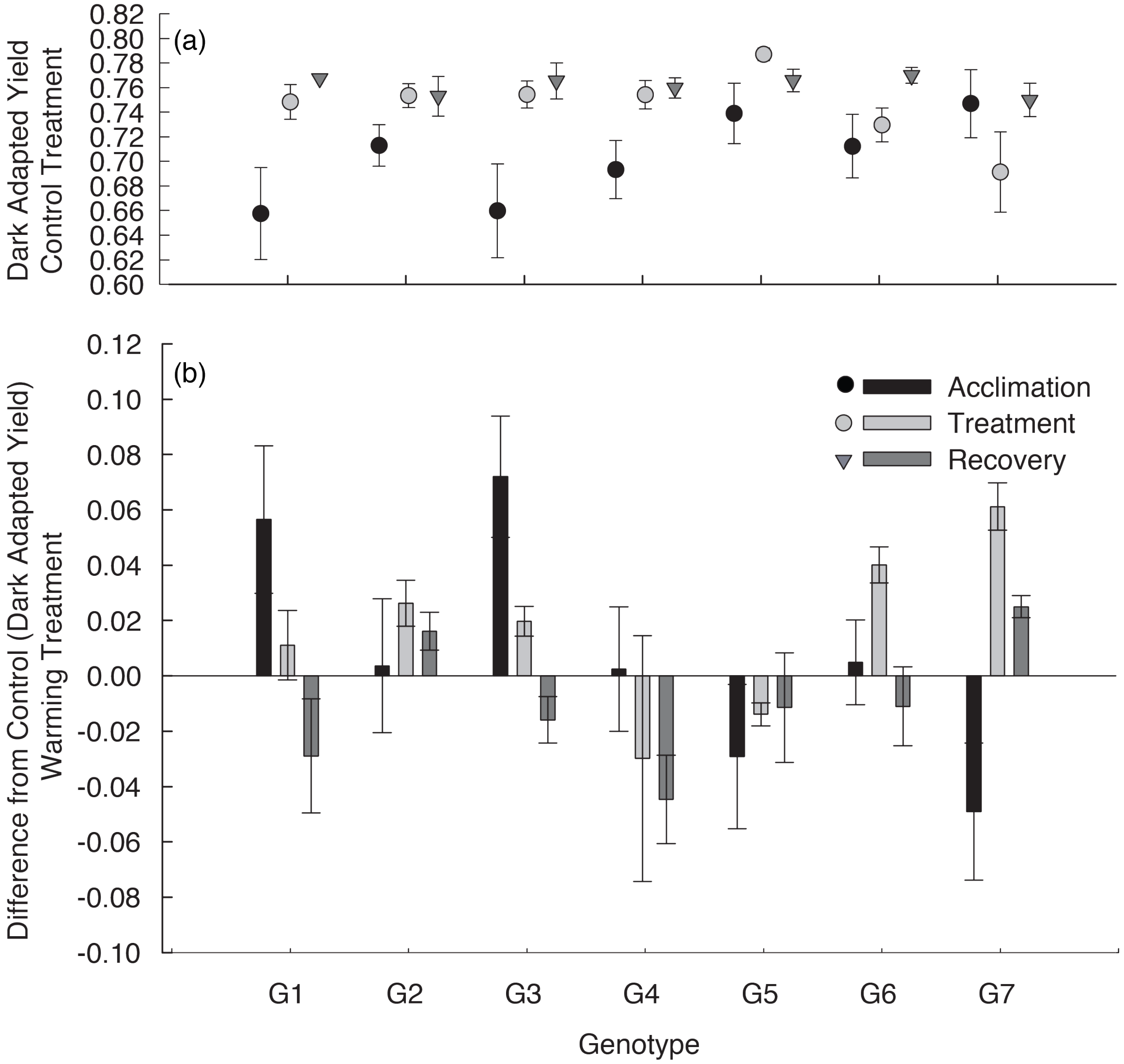

Figure H in S2. Dark adapted yield (F_V/F_m measured by Pulse Amplitude Modulation) for 7 distinct *Zostera marina* genotypes over time.

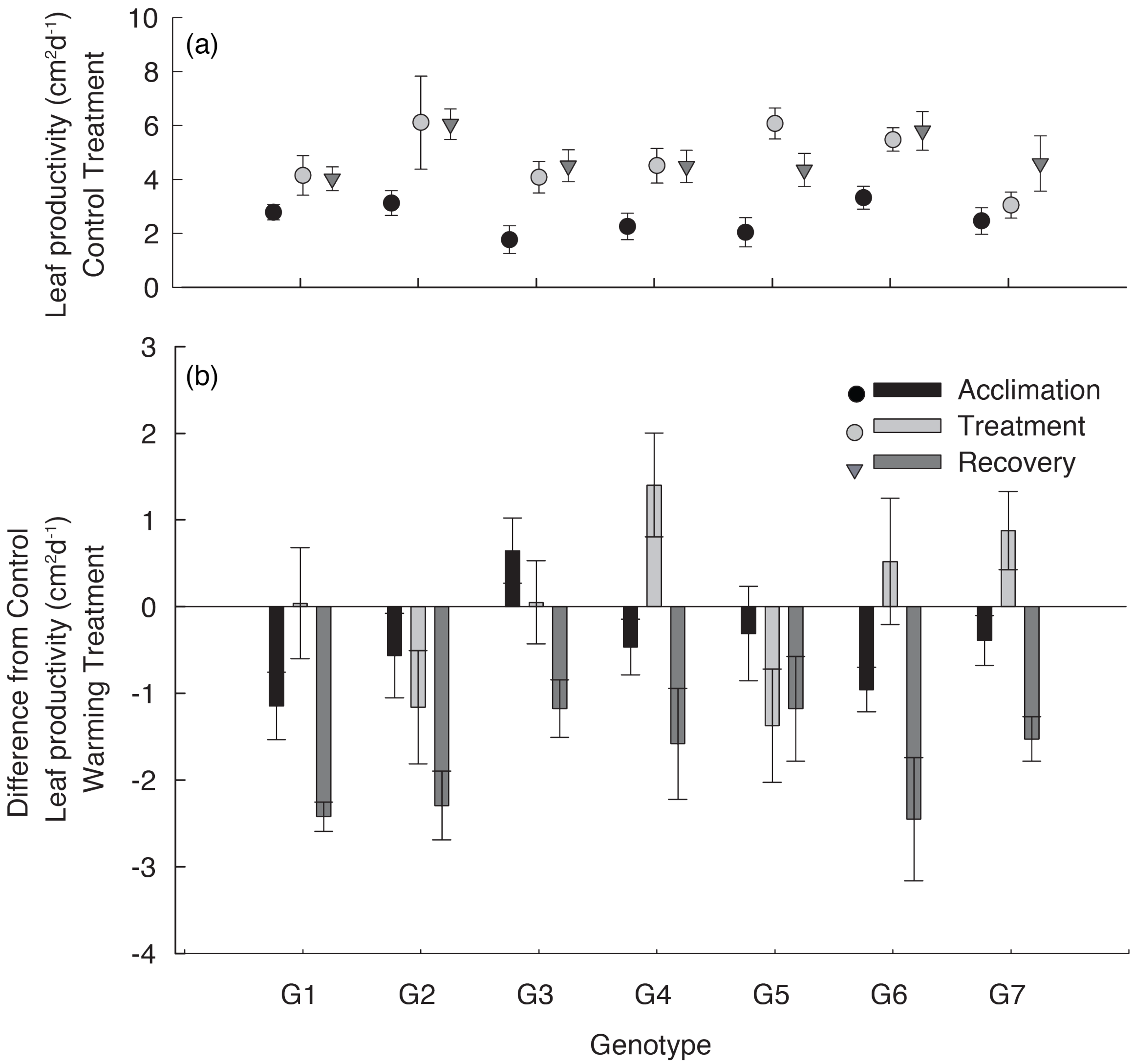

Figure I in S2. Leaf growth, measured as leaf area produced, for 7 distinct *Zostera marina* genotypes over time.

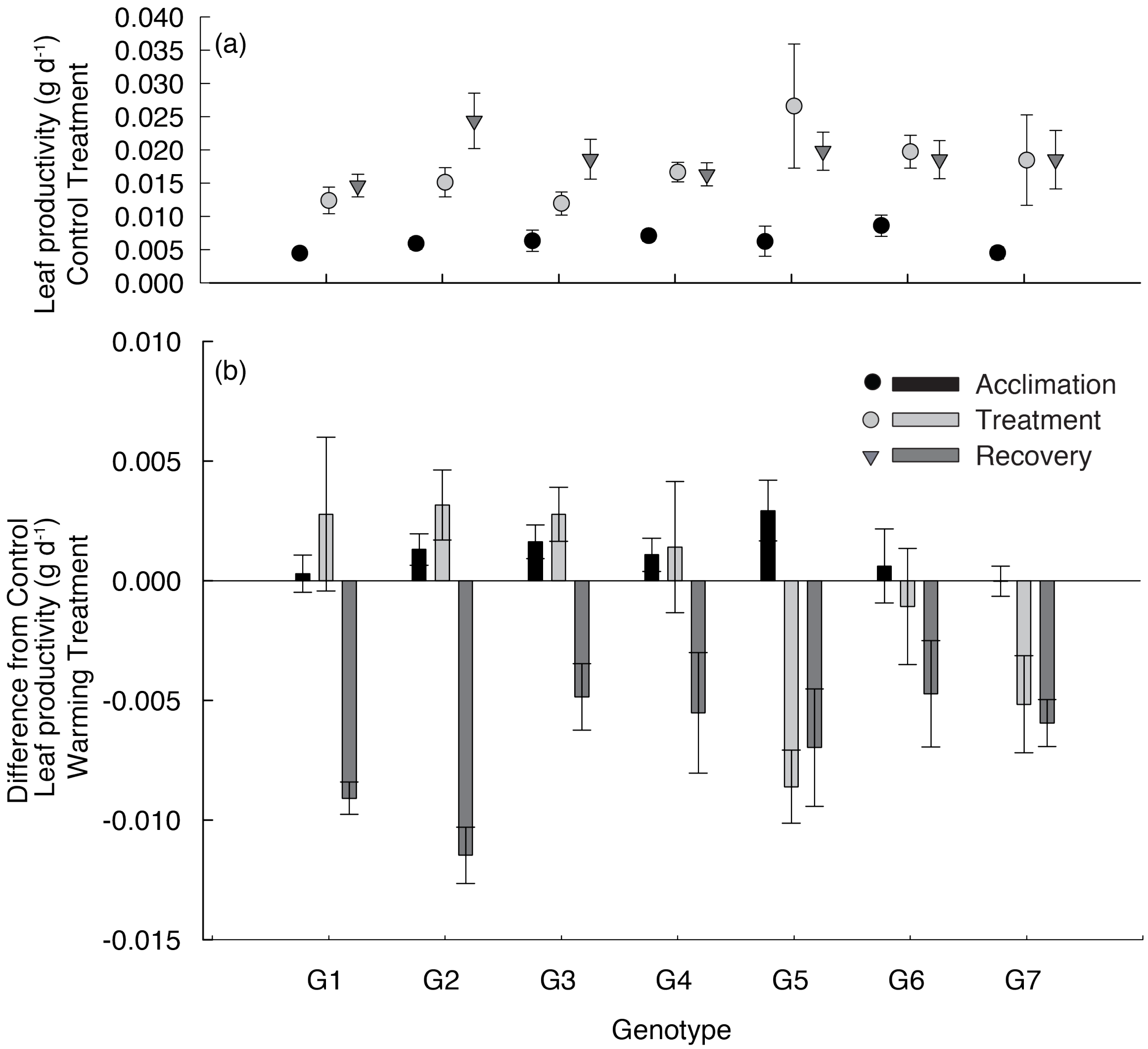

Figure J in S2. Leaf growth, measured as biomass produced, for 7 distinct *Zostera marina* genotypes over time.

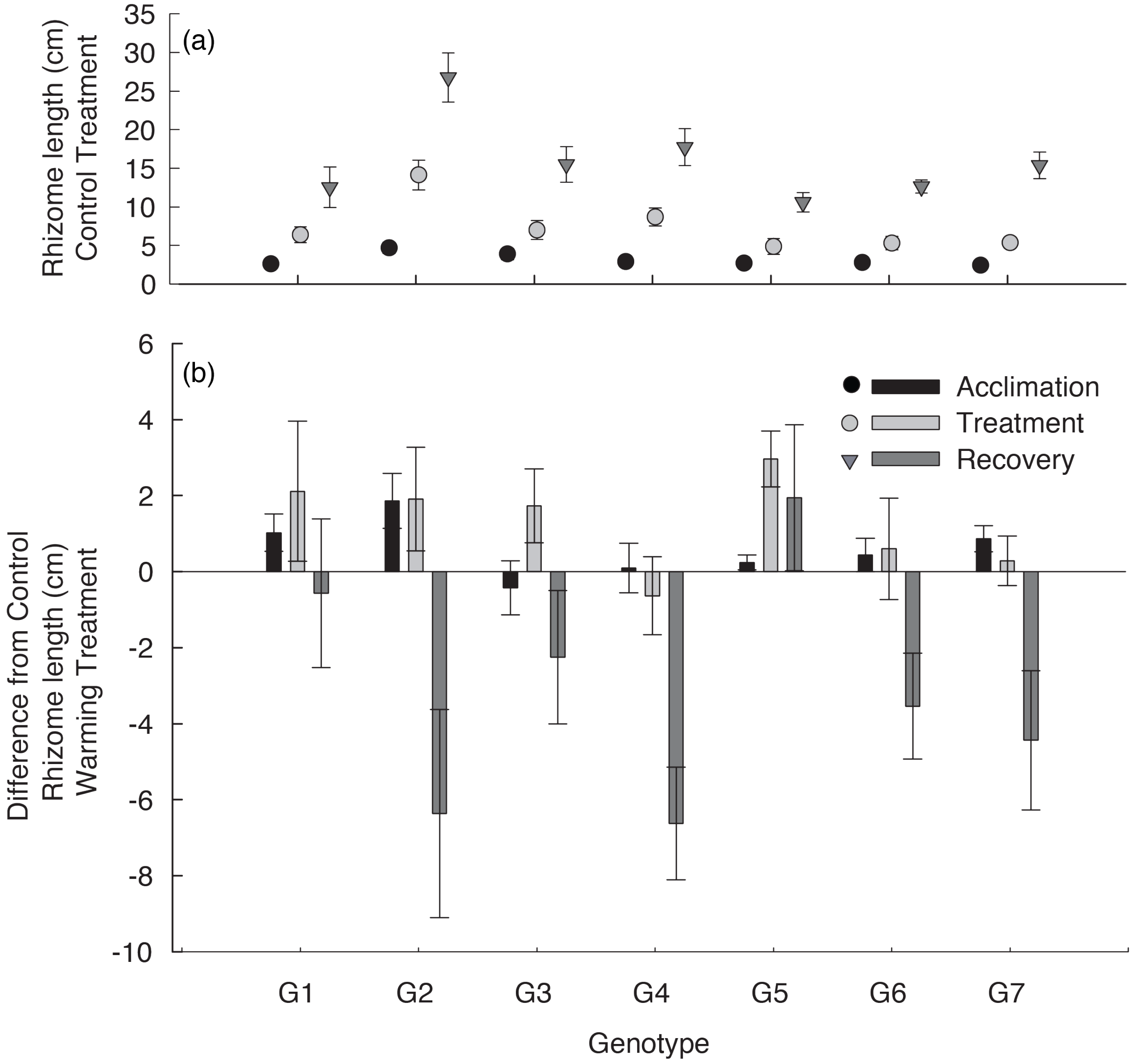

Figure K in S2. Cumulative rhizome elongation for 7 distinct *Zostera marina* genotypes over time.

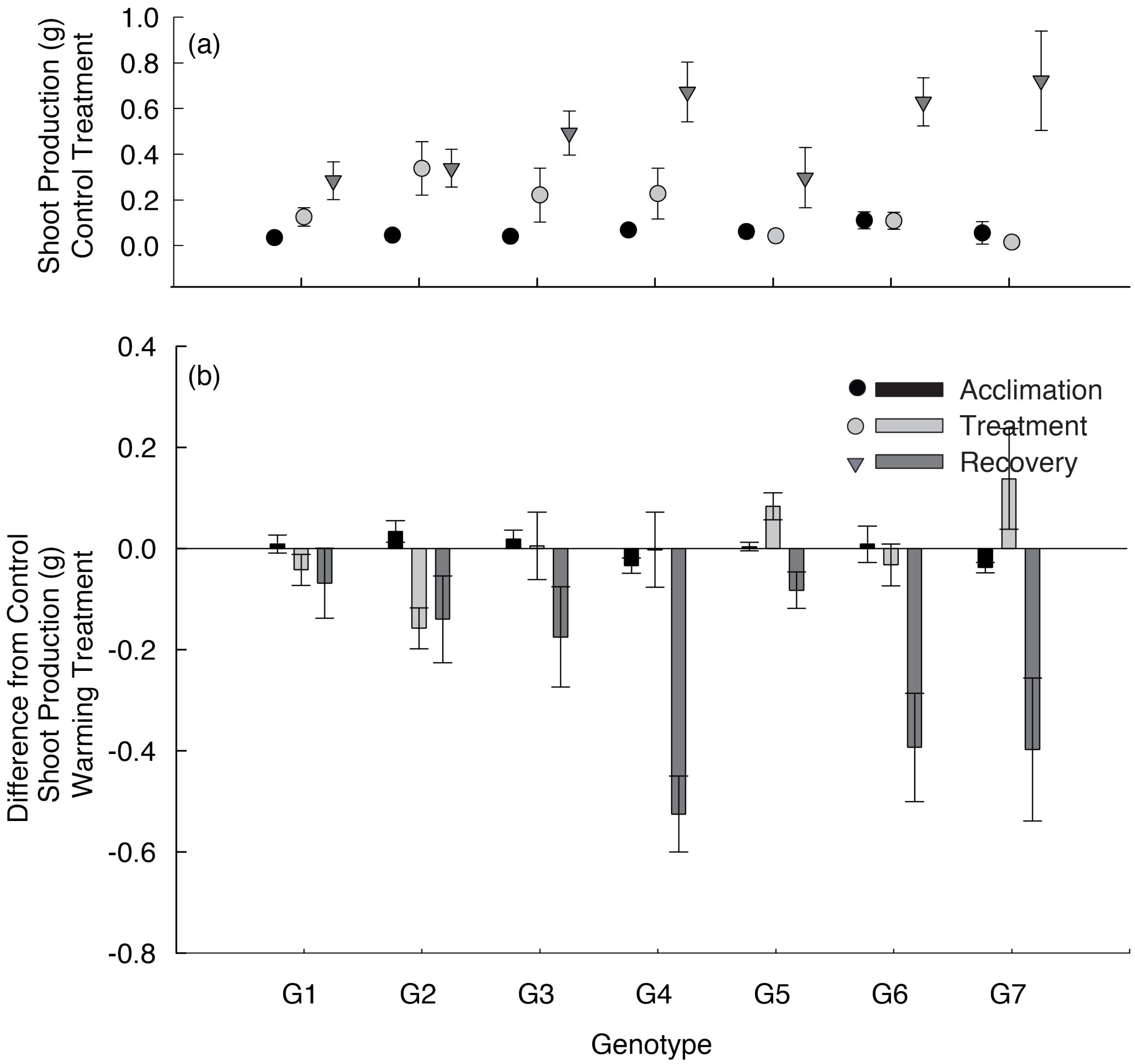

Figure L in S2. Cumulative shoot production (measured as biomass) for 7 distinct

Zostera marina genotypes over time.
